# Optimal strategy for delirium detection in older patients admitted to intensive care unit after non-cardiac surgery

**DOI:** 10.3389/fsurg.2023.1095329

**Published:** 2023-03-27

**Authors:** Kun Wang, Xian Su, Jia-Hui Ma, Dong-Xin Wang

**Affiliations:** ^1^ Department of Anesthesiology, Peking University First Hospital, Beijing, China; ^2^ Department of Outcomes Research, Outcomes Research Consortium, Cleveland, OH, United States

**Keywords:** delirium, cognitive fuction, elderly, noncardiac surgery, CAM-ICU (confusion assessment method for the intensive care unit)

## Abstract

**Background:**

Delirium detection is challenging due to the fluctuating nature and frequent hypoactive presentation. This study aimed to determine an optimal strategy that detects delirium with higher sensitivity but lower effort in older patients admitted to the intensive care unit (ICU) after surgery.

**Methods:**

This was a secondary analysis of the database from a randomized trial. Seven hundred older patients (aged ≥65 years) who were admitted to the ICU after elective noncardiac surgery were enrolled. Delirium was assessed with the Confusion Assessment Method for the ICU (CAM-ICU) twice daily during the first 7 days postoperatively. The sensitivity of different strategies in detecting delirium were analyzed and compared.

**Results:**

Of all enrolled patients, 111 (15.9%; 95% CI: 13.3% to 18.8%) developed at least one episode of delirium during the first 7 postoperative days. Among patients who developed delirium, 60.4% (67/111) had their first delirium onset on postoperative day 1, 84.7% (94/111) by the end of day 2, 91.9% (102/111) by the end of day 3, and 99.1% (110/111) by the end of day 4. Compared with delirium assessment twice daily for 7 days, twice-daily measurements for 5 days detected 100% of delirium patients with 71% efforts; twice-daily measurements for 4 days detected 99% (95% CI: 94% to 100%) of delirium patients with 57% efforts; twice-daily assessment for 3 days detected 92% (95% CI: 85% to 96%) of delirium patients with only 43% efforts.

**Conclusions:**

For older patients admitted to the ICU after elective noncardiac surgery, it is reasonable to detect delirium with the CAM-ICU twice daily for no more than 5 days, and if the personnel and funds are insufficient, 4 days could be sufficient.

## 1. Introduction

Delirium is an acutely occurred and short-lived syndrome, characterized by fluctuating changes in attention, level of consciousness, and cognitive function (1). It is a common complication after major surgery, especially in older patients (2). According to a recent systematic review, the reported incidence ranged from 4% to 46% in patients aged ≥60 years following noncardiac surgery (3). The occurrence of postoperative delirium is associated with increased morbidity and mortality (4), prolonged stays in the intensive care unit (ICU) and hospital, and worsened functional recovery (5, 6). Studies showed that postoperative delirium mostly occurs in the first few days (7–9). Various diagnostic tools, such as the Confusion Assessment Method (CAM) and the CAM for the intensive care unit (CAM-ICU), have been introduced and validated to facilitate delirium screening (10–12).

The detection of delirium can be challenging due to the fluctuating nature and frequent hypoactive presentation. Interview-based methods, i.e., delirium assessed by trained interviewers during brief encounters, is frequently used in clinical studies and daily practice (13). However, it is still unknown how often should the assessments be done and how long should the evaluation period be. A previous study reported that daily assessment is not enough because cases occurring during night-shift might be missed (14). In a recent study of 788 patients following cardiac surgery, delirium was detected more often in the mornings than evenings, and CAM-ICU assessment twice daily for 4 days detected an estimated 97% of postoperative delirium (15).

In this analysis, we aimed to evaluate the effect of different strategies in detecting delirium during the first 7 days after non-cardiac surgery, and to determine the most favorable strategy that detected delirium with high sensitivity and low effort.

## 2. Methods

### 2.1. Study design

This was a *post hoc* analysis of data collected during a randomized trial. The underlying trial was conducted in two affiliated hospitals of Peking University from August 17, 2011 to November 20, 2013 (www.chictr.org.cn, number ChiCTR-TRC-10000802) (5). The protocol for this analysis was approved by the Biomedical Research Ethics Committee of Peking University First Hospital [2022(407) on September 21, 2022; No. 8 Xishiku Street, Beijing 100034, China; Chairperson Prof. Yan-Yan Yu]; informed consent was waived since all analysis was performed on de-identified data without any further patient/family member contact.

### 2.2. Participants

We enrolled patients aged ≥65 years who were admitted to the ICU after elective noncardiac surgery. We excluded those who met the following criteria: refused to participate; preoperative history of schizophrenia, epilepsy, Parkinson's disease, or myasthenia gravis; inability to communicate because of coma, severe dementia or language barriers before surgery; brain injury or neurosurgery; preoperative left ventricular ejection fraction (LVEF) <30%, sick sinus syndrome, severe sinus bradycardia (<50 beats per minute), or second-degree or higher atrioventricular block without pacemaker; severe liver dysfunction (Child-Pugh C grade); severe renal dysfunction (preoperative renal replacement therapy); or expected survival <24 h (5).

### 2.3. Procedures and outcomes

In the underlying trial, the enrolled patients were randomly assigned to receive intravenous infusion of either placebo (normal saline) or dexmedetomidine (at a rate of 0.1 μg/kg/h) from ICU admission on the day of surgery until 8:00 am on the first day after surgery (5).

Postoperative delirium was assessed with the CAM-ICU (11, 16) by investigators who had been trained by a psychiatrist. The CAM-ICU detects four features of delirium including (1) acute onset of mental status changes or a fluctuating course, (2) inattention, (3) disorganized thinking, and (4) altered level of consciousness. Patients showing features of (1) and (2), with either (3) or (4), were diagnosed as having delirium. The Chinese verison CAM-ICU had been validated in the ICU setting (11, 16). We have considerable experience in assessing delirium with the CAM-ICU (17, 18).

Delirium assessment was performed twice daily, i.e., from 8:00 to 10:00 am and from 18:00 to 20:00 pm, during the first 7 postoperative days or until hospital discharge. Before each delirium assessment, sedation and agitation was evaluated with the Richmond Agitation Sedation Scale (RASS); the score ranges from −5 [unarousable] to +4 [combative] and 0 indicates alert and calm (19). Deeply sedated or unarousable patients (RASS score −4 or −5) were marked as comatose and not assessed for delirium. Delirium was assessed for those with a RASS score from −3 to +4. Investigators performing delirium assessment had been trained by a psychiatrist before initiating the trial.

### 2.4. Statistical analysis

Continuous variables with normal distribution were analyzed using the independent samples *t*-test. Continuous variables with non-normal distribution and ranked data were analyzed using the Mann–Whitney *U* test. Categorical variables were analyzed using the chi-square test or Fisher's exact test. We used frequency tables to describe the number (%) of delirium assessments completed each morning and afternoon on postoperative days 1–7, and the proportion of patients with detected delirium at different times and frequencies of assessment. Kaplan–Meier estimators were used to analyze time to first-onset delirium over postoperative days 1–7 with 3 different evaluation strategies: morning and afternoon assessments, morning only assessments, and afternoon only assessments. McNemar's test for paired proportions was used to compare morning and evening delirium detection. We used the Wilson score method to estimate the 95% CIs for the proportion of postoperative delirium cases detected for each evaluation strategy. SPSS statistical software version 25.0 was used for all analyses.

## 3. Results

A total of 700 patients were enrolled in the underlying trial. The average age of enrolled patients was 74 years, with 60.4% were male. Patients who experienced delirium were older, had lower body mass index, suffered more previous stroke, had lower preoperative serum albumin, were more frequently admitted to ICU with intubation, and received less low-dose dexmedetomidine when compared with those who did not (Table 1).

**Table 1:** Baseline and perioperative data.

Items	All patients (*n* = 700)	Without delirium (*n* = 589)	With delirium (*n* = 111)	*P* value
Age (year)	74.3 ± 6.8	74.0 ± 6.6	76.2 ± 7.8	**0** **.** **001**
Male sex	423 (60.4%)	365 (62.0%)	58 (52.3%)	0.055
Body mass index (kg/m^2^)	23.7 ± 3.9	24.0 ± 4.2	22.5 ± 4.2	**<0**.**001**
Preoperative comorbidity				
Hypertension	446 (63.7%)	378 (64.2%)	68 (61.3%)	0.558
Coronary heart disease	232 (33.1%)	200 (34.0%)	32 (28.8%)	0.293
Stroke	161 (23.0%)	126 (21.4%)	35 (31.5%)	**0**.**020**
Smoking^a^	176 (25.1%)	143 (24.3%)	33 (29.7%)	0.225
Diabetes	190 (27.1%)	163 (27.7%)	27 (24.3%)	0.467
Liver dysfunction^b^	19 (2.7%)	14 (2.4%)	5 (4.5%)	0.205
Renal dysfunction^c^	35 (5.0%)	26 (4.4%)	9 (8.1%)	0.101
Preoperative laboratory test				
Hematocrit (%)	36.4 ± 5.7	36.6 ± 5.6	35.4 ± 5.9	0.051
Albumin (g/L)	38.1 ± 5.2	38.5 ± 5.0	36.0 ± 6.0	**<0**.**001**
ASA classification				0.057
Class II	398 (56.9%)	344 (58.4%)	54 (48.6%)	
Class III	302 (43.1%)	245 (41.6%)	57 (51.4%)	
Duration of anesthesia (min)	314 ± 146	310 ± 141	333 ± 169	0.132
Type of anesthesia				0.361
General	578 (82.6%)	483 (82.0%)	95 (85.6%)	
Epidural-general	122 (17.4%)	106 (18.0%)	16 (14.4%)	
Blood transfusion	114 (16.3%)	90 (15.3%)	24 (21.6%)	0.097
Site of surgery				0.073
Intraabdominal	475 (67.9%)	410 (69.6%)	65 (58.6%)	
Intrathoracic	120 (17.1%)	97 (16.5%)	23 (20.7%)	
Spinal-extremital	36 (5.1%)	26 (4.4%)	10 (9.0%)	
Superficial-transurethral	69 (9.9%)	56 (9.5%)	13 (11.7%)	
Duration of surgery (min)	228 ± 137	225 ± 131	244 ± 165	0.257
Use of PCA				0.594
None	73 (10.4%)	61 (10.4%)	12 (10.8%)	
Intravenous^d^	516 (73.7%)	431 (73.2%)	85 (76.6%)	
Epidural^e^	111 (15.9%)	97 (16.5%)	14 (12.6%)	
ICU admission with intubation	382 (54.6%)	305 (51.8%)	77 (69.4%)	**<0**.**001**
Low-dose dexmedetomidine	350 (50%)	318 (54.0%)	32 (28.8%)	**<0**.**001**
Additional sedatives and/or analgesics within 7 days				
Propofol	357 (51%)	290 (49.2%)	67 (60.4%)	0.032
Midazolam	58 (8.3%)	45 (7.6%)	13 (11.7%)	0.153
Morphine	201 (28.7%)	166 (28.2%)	35 (31.5%)	0.474
Flurbiprofen axetil	226 (32.3%)	188 (31.9%)	38 (34.2%)	0.632

Data are mean ± SD or *n* (%). *P* values in bold indicate <0.05.

ASA, American Society of Anesthesiologists; PCA, patient-controlled analgesia; ICU, intensive care unit.

^a^ Daily smoking of cigarettes more than half a pack for two years or more.

^b^ Alanine aminotransferase and/or aspartate aminotransferase higher than 5 times of the normal upper limit.

^c^ Serum creatinine level ≥177 μmol/L.

^d^ Established with 100 ml of 0.5 mg/ml morphine or 1.25 μg/ml sufentanil, programmed to deliver a 2 ml bolus with a lockout interval of 6–10 min and a background infusion of 1 ml/h.

^e^ Established with 250 ml of 0.12% ropivacaine plus 0.5 μg/ml sufentanil, programmed to deliver a 2 ml bolus with a lockout interval of 20 min and a background infusion of 4 ml/h.

Of all enrolled patients, 111 (15.9%; 95% CI: 13.3% to 18.8%) developed at least one episode of delirium during the first 7 postoperative days. Among these, 60.4% (67/111) had their first delirium onset on postoperative day 1, 84.7% (94/111) by the end of day 2, 91.9% (102/111) by the end of day 3, 99.1% (110/111) by the end of day 4, and 100% (111/111) by the end of day 5 (Table 2).

**Table 2:** First delirium event for patients (*n* = 111) with any positive delirium during the first 7 postoperative days.

Day	Time	*N* (%) with initial positive assessment at given time	Cumulative *n* (%) by given time
1	AM	40 (36.0%)	40 (36.0%)
	PM	27 (24.3%)	67 (60.4%)
2	AM	11 (9.9%)	78 (70.3%)
	PM	16 (14.4%)	94 (84.7%)
3	AM	4 (3.6%)	98 (88.3%)
	PM	4 (3.6%)	102 (91.9%)
4	AM	5 (4.5%)	107 (96.4%)
	PM	3 (2.7%)	110 (99.1%)
5	AM	1 (0.9%)	111 (100%)
	PM	0 (0%)	111 (100%)
6	AM	0 (0%)	111 (100%)
	PM	0 (0%)	111 (100%)
7	AM	0 (0%)	111 (100%)
	PM	0 (0%)	111 (100%)
Total		111 (100%)	111 (100%)

Data are *n* (%).

**Figure 1:**
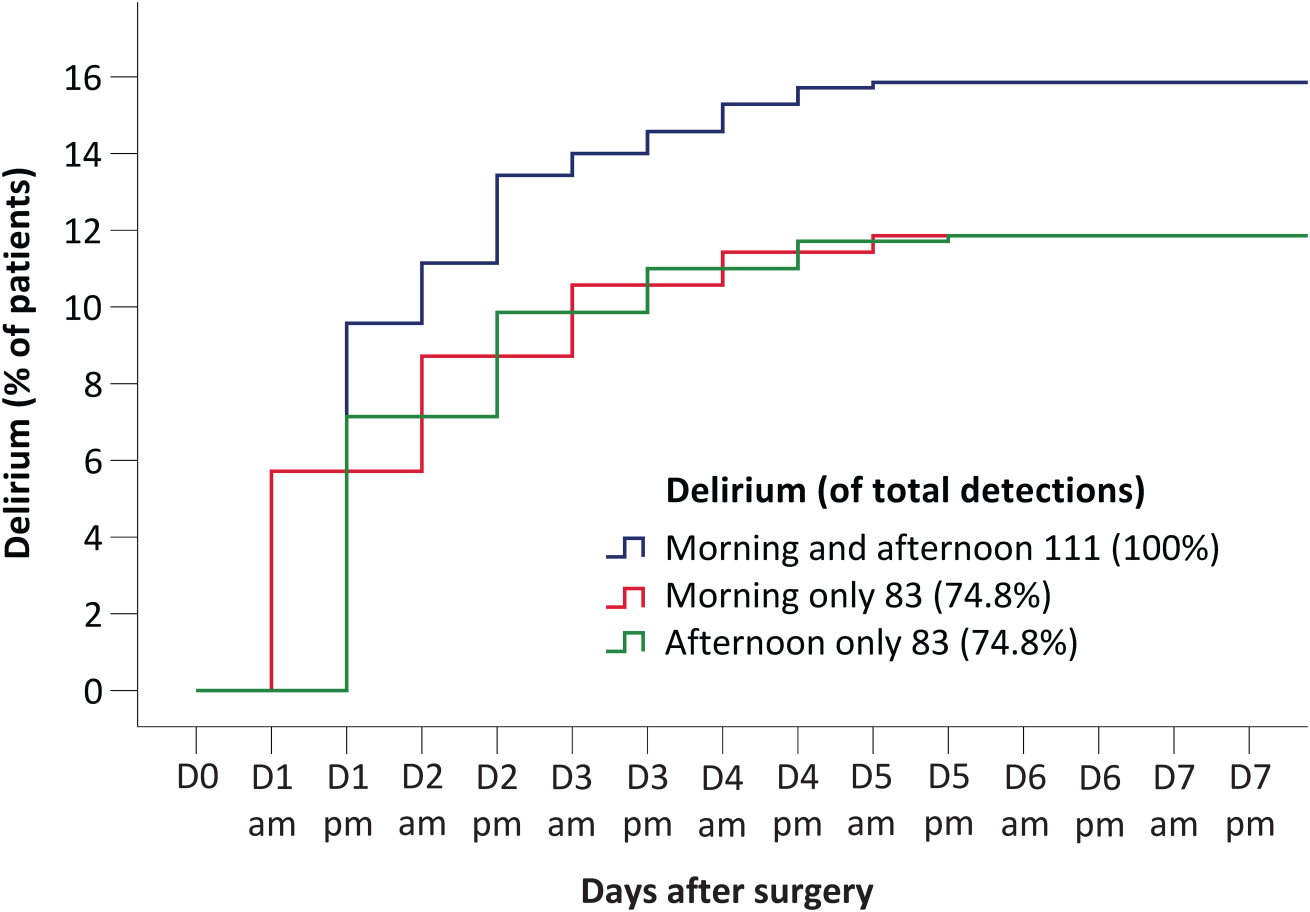
Time to onset of delirium using morning and afternoon assessments (blue curve), morning assessments only (red curve), and afternoon assessments only (green curve). Results to the right of figure legends indicate the number and fraction of the 111 detected delirium cases identified with each strategy.

We compared the sensitivity of different strategies in detecting first postoperative delirium compared with twice-daily assessments for 7 days (Table 3). There was no significant difference in the sensitivity of delirium detection with morning only or afternoon only assessments (Figure 1). Both of the above strategies detected 83 (11.9% of 700) delirium patients during the first 7 postoperative days. Twice-daily measurements for 4 days detected 99% (95% CI: 94% to 100%) of delirium patients with 57% efforts. Similarly, twice-daily assessment for 3 days detected 92% (95% CI: 85% to 96%) of delirium patients with only 43% efforts.

**Table 3:** Various evaluation strategies for detecting postoperative delirium.

	Day 1	Day 2	Day 3	Day 4	Day 5	Day 6	Day 7	Total number of visits	Patients with first positive evaluation (*n* = 700)	Sensitivity of each strategy (95% CI)^a^
AM	X	X	X	X	X	X	X	14	111 (15.9%)	100%
PM	X	X	X	X	X	X	X			
AM	X	X	X	X	X	X	X	11	111 (15.9%)	100%
PM	X	X	X	X						
AM	X	X	X	X	X	X	X	10	109 (15.6%)	98% (94%, 99%)
PM	X	X	X							
AM	X	X	X	X	X	X	X	9	106 (15.1%)	95% (89%, 98%)
PM	X	X								
AM	X	X	X	X	X	X	X	8	96 (13.7%)	86% (79%, 92%)
PM	X									
AM	X	X	X	X	X	X	X	7	83 (11.9%)	75% (66%, 82%)
PM										
AM	X	X	X	X	X			10	111 (15.9%)	100%
PM	X	X	X	X	X					
AM	X	X	X	X				8	110 (15.7%)	99% (94%, 100%)
PM	X	X	X	X						
AM	X	X	X					6	102 (14.6%)	92% (85%, 96%)
PM	X	X	X							
AM	X	X						4	94 (13.4%)	85% (77%, 90%)
PM	X	X								
AM								7	83 (11.9%)	75% (66%, 82%)
PM	X	X	X	X	X	X	X			

^a^ Sensitivity of various evaluation strategies for detecting postoperative delirium compared with twice-daily assessments for 7 days. Clopper-Pearson confidence interval for a binomial proportion.

## 4. Discussion

In the present study, delirium occurred in 15.9% of our patients during the first seven days after non-cardiac surgery. The rate was relatively lower than previously reported incidence (24.4% to 44.5%) in similar patient populations (20, 21). This could be explained by the fact that half of our patients received low-dose dexmedetomidine infusion during the night after surgery, a regimen that has been proved effective in decreasing delirium in high-risk patients (5, 22, 23). Another possible reason was that multiple non-pharmacological measures have been widely applied in clinical practice to prevent delirium (24, 25). When patients given dexmedetomidine were excluded, the incidence of delirium (22.6%) was close to the reported range.

Our results showed that delirium mainly occurred early after surgery, i.e., 91.9% of new-onset delirium developed within the first three postoperative days in our patients. This is in line with other studies (8, 26). Similar results were also reported in recent trials. For example, 89% of all delirious cases occurred within 3 days after cardiac surgery (7), and that after noncardiac surgery was 88% (27). The phenomenon could be attributed to the fact that surgery-related responses including the degree of inflammation and the secretion of neuroendocrine hormone, which play important roles in the pathogenesis of delirium, peak in the early postoperative period (17, 28–30).

Delirium has a fluctuating nature and is frequently presented in a hypoactive form (31, 32). It is not surprising that many delirious cases, up to 76%, were miss diagnosed in the settings of emergency department, palliative care unit, and ICU (33–35). Delirium detection can be improved with frequent monitoring, and early treatment of the underlying condition can improve outcome (36). However, redundant evaluations require more manpower and can be costly. The commonly used delirium evaluation strategy is twice daily after surgery for several days. Limiting the number of delirium assessments while maintaining high sensitivity would help to make the research work cheaper and more practical.

During the analysis, we designed various evaluation strategies to simplify the original one that assessed delirium twice daily for 7 days after surgery. We found that CAM-ICU assessments twice daily for 5 postoperative days detected 100% delirium but required 29% less evaluation, while CAM-ICU assessment twice daily for 4 days detected 99% delirium but required 43% less evaluation. It is thus reasonable to design studies with no more than 5 days of delirium assessments, and if the personnel and funds of the study are insufficient, 4 days could be sufficient. A previous study reported that delirium was detected more often in the morning than in the evening (15). However, we did not find difference in detecting delirium between morning only and afternoon only assessments, possibly due to different patient population and improved nighttime environment and care in the ICU. Both methods detected only 75% delirium within 7 days and are therefore not recommended.

The advantages of our study included that the investigators who performed delirium assessment had been trained by a psychiatrist and that delirium assessment was performed twice daily for 7 consecutive days. These helped us to detect most delirium cases. There are also some limitations. Firstly, while investigators had been trained for delirium assessment, the inter-investigator differences were hardly avoidable. Secondly, we enrolled patients following various kinds of non-cardiac surgeries. This increases the generalizability of our study but increases the complexity in explaining the results. Thirdly, all patients included in the underlying trial were admitted to the ICU after noncardiac surgery. Whether our results can be extrapolated to non-ICU patients requires further investigation.

## 5. Conclusions

In older patients admitted to the ICU after elective noncardiac surgery, 60.4% of all delirium detected over 7 days occurred during the first postoperative day and 91.2% occurred within the first 3 postoperative days. Compared with CAM-ICU assessment twice daily for 7 days, a twice-daily assessment for 5 days detected 100% delirium with 29% less effort, while a twice-daily assessment for 4 days detected 99% delirium with 43% less effort. Delirium assessments should therefore be performed twice daily for at least 4 initial postoperative days.

## Data Availability

The raw data supporting the conclusions of this article will be made available by the authors, without undue reservation.
